# Every Day’s a New Day: A Captain’s Reflection on a Losing Season

**DOI:** 10.3390/sports6040115

**Published:** 2018-10-10

**Authors:** Fraser Carson, Julia Walsh

**Affiliations:** Centre for Sport Research, School of Exercise and Nutrition Sciences, Deakin University, Geelong, VIC 3220, Australia; Julia.walsh@deakin.edu.au

**Keywords:** leadership, elite sport, basketball team, coping, social phenomenological analysis

## Abstract

Being captain of any team is a significant and prestigious position. In elite sport, the captain plays a vital role in both team and organisational leadership. To date there has been minimal research investigating captaincy, and none assessing the impact of regularly losing performances. A captain of a women’s national basketball league team participated in an in-depth, semi-structured interview reflecting on her experience during a losing season. Following Schutz’s (1967) framework, a social phenomenological analysis approach was taken, with seven higher-order themes emerging: being captain; relationship with coaching staff; relationship with teammates; team development; stressors; stress management; and self. Results suggest that while poor results affect all team members, there are increased levels of stress for the captain. The captain is expected to lead by example and avoid external expression of negativity. Further strains are placed on the captain, as they are the conduit between coaching and playing groups. As a result, the captain needs to have good support networks, from a variety of sources, to cope and minimise the impact on personal performance.

## 1. Introduction

Captains play a vital leadership role within team sport settings. They are a formal, designated leader of the playing group and an important liaison between the team members and the coaching staff [1]. Most captains are selected based on their fit for the team [2], and their role is perceived to be highly important for team success in professional sport [3].

Despite increased research on leadership in sport settings, Cotterill and Cheetham [3] noted there was still limited research on the captain, their role, and the challenges they face, particularly at elite level. This may be due to the complexity of the captain’s role, which has lacked clarity and varies greatly between teams and sports. Mosher [1] identified three key components to the captain’s role: (1) to lead the team in all areas; (2) be the link between the players and the coaching staff, and vice-versa; and (3) represent the team at official events. Fransen et al. [4] expand the leadership role of the captain into four major areas: (1) Task—to focus team goals and behaviours to reach team objectives; (2) Motivation—to encourage and engage teammates to perform at their peak; (3) Social—promoting harmony and cohesion within the team; and (4) External—to communicate with all outside influences. However, the role of the captain goes beyond this to include a vast array of responsibilities and functions [3].

Becoming the captain is significant. It is a position of recognition and generally a reward for service to the team. Within high school settings, Voelker et al. [5] found the experience to be positive. The role provides new opportunities to grow, including development of teamwork and social skills [6]. Within elite sport settings the captain is a position of privilege; a privilege limited to few [7].

Like any leadership position, captaincy is challenging [5]. The captain simultaneously faces a number of stressors and high demands, with most captains confronting several performance and organisational pressures [7]. The captain needs to be professional at all times, regardless of the situation [1]. They need to lead by example [8]. However, many captains receive minimal support on how become an effective leader [5].

Given the minimal current knowledge into formal leadership in sport, especially in relation to the captain’s role in a women’s professional sports organisation, the purpose of this study was to examine this unique, lived experience of the captain. Further, as none of the research has related to the experience during a losing season (i.e., less than 50% win record across the regular season), the outcome sought from this study was to provide greater insight into the challenges, stressors, and benefits of being a captain under intense pressure to improve performance.

## 2. Materials and Methods

### 2.1. Design

A social phenomenological [9] approach was taken as both a philosophical framework and methodology for this study, as it allowed for a descriptive and interpretive exploration of the captain’s subjective experience. This approach takes the view that people involved are able to attribute meaning to the situation and make judgements, emphasising the spatial and temporal aspects of the experience and social relationships. Schutz [10] encouraged examination of the subjective viewpoint, rather than being constructed by the research, stating “*the safeguarding of the subjective point of view is the only but sufficient guarantee that the world of social reality will not be replaced by a fictional nonexisting world constructed by the scientific observer*” (p. 271). In doing so, Schutz [9] provided two methods of interpretive understanding: (1) the process by which people make sense of the everyday world; and (2) generating “ideal types” to explain the phenomenon under investigation. This study addressed the second of these by utilising a data-driven inductive analysis approach [11].

To demonstrate rigour within the social phenomenological framework, Schutz [12] described three essential postulates to follow (pp. 43–44):

1. The postulate of logical consistency—developing the highest degree of clarity of the conceptual framework and method applied, and conform to the principles of formal logic.
2. The postulate of subjective interpretation—the model must be grounded in the subjective meaning the action had for the “actor”.
3. The postulate of adequacy—there must be consistency between the researcher’s constructs and typifications and those found in common-sense experience. The model must be understandable to the actor and must be able to explain an action appropriately.

The first postulate of logical consistency involves in-depth planning and careful attention to the phenomenon being studied, followed by the production of useful results. The step-by-step process of analysis described for this study provides transparency of the research process to enhance the methodological rigour. The second postulate, subjective interpretation, is concerned with preserving the subjective viewpoint of the participant; this is achieved in this study by privileging the participant’s voice through extensive use of quotations from the raw data provided within the results. The third postulate of adequacy focused on validating the participant’s responses, which is addressed by member checking of the full interview transcript and the final manuscript.

Ethical approval was provided from the University Ethics Committee at the authors’ institution, and the participant provided written and verbal consent prior to the interview, for both research and publication purposes.

### 2.2. Participant

The participant was appointed the captain of a women’s national basketball league team prior to the 2016/2017 season. The league is ranked within the top three competitions in the world. She had been exposed to previous leadership roles and shared leadership roles in women’s professional sport. In the 2016 season she transitioned from a shared captaincy to sole captain due to changes in team structure. To provide some confidentiality, and due to the small population from which she was recruited, no further demographic information is provided.

### 2.3. Procedure

The participant was interviewed, utilising a semi-structured interview approach that encompassed reflecting on her experience as captain during the season and discussion on influencing factors throughout the season. Consistent with social phenomenological approaches, the interview script was used to guide discussion and initiate reflection, rather than a prescriptive process. The interview covered several areas including: pre-season preparation; captain-coach relations; captain-team relations; early season success; coping with losing; team cohesion; response to missing post-season; and reflections on critical incidents. Examples of questions were: “How did being made captain influence your approach to the new season?”; “Describe your relationship with the coaching staff during the losing period? Is this different as a captain as opposed to team member”; and “How does the multiple losses impact you as the leader of the team?”. The interview lasted 48 min and was recorded and transcribed to produce an accurate and exact record of the discussion.

Following Lincoln and Guba’s [13] guidelines, the researchers employed a number of strategies to improve the methodological rigour and develop trustworthiness of data. Credibility was achieved in five ways: (a) triangulation via the methods and multiple analyst review; (b) peer debriefing; (c) negative case analysis; (d) referential adequacy; and (e) member checking. Transferability was achieved by providing a thick description within the results section.

To increase the trustworthiness of the data, member checking was conducted with the participant following an initial draft of the research findings and again when the full version of the manuscript had been completed. At both stages, the participant was asked to corroborate the interpretations made. Conceivability was achieved by having an audit trail of the data and data analysis available, and through triangulation.

### 2.4. Data Analysis

The audio recording and written transcript were listened to and read on multiple occasions to obtain familiarity with the participant’s account. Preliminary notes, observations, and reflections related to the interview were made in the left-hand margin of the transcript and significant comments highlighted in the text. Following this, connections and emergent themes were identified, and checks were made to compare notes to specific stages of the interview to ensure these themes were accurate. The themes were then combined with direct quotations from the transcript to ensure richness of the analysis. This process allowed for these lower-order themes to be clustered into higher-order themes.

## 3. Results

**Figure 1:**
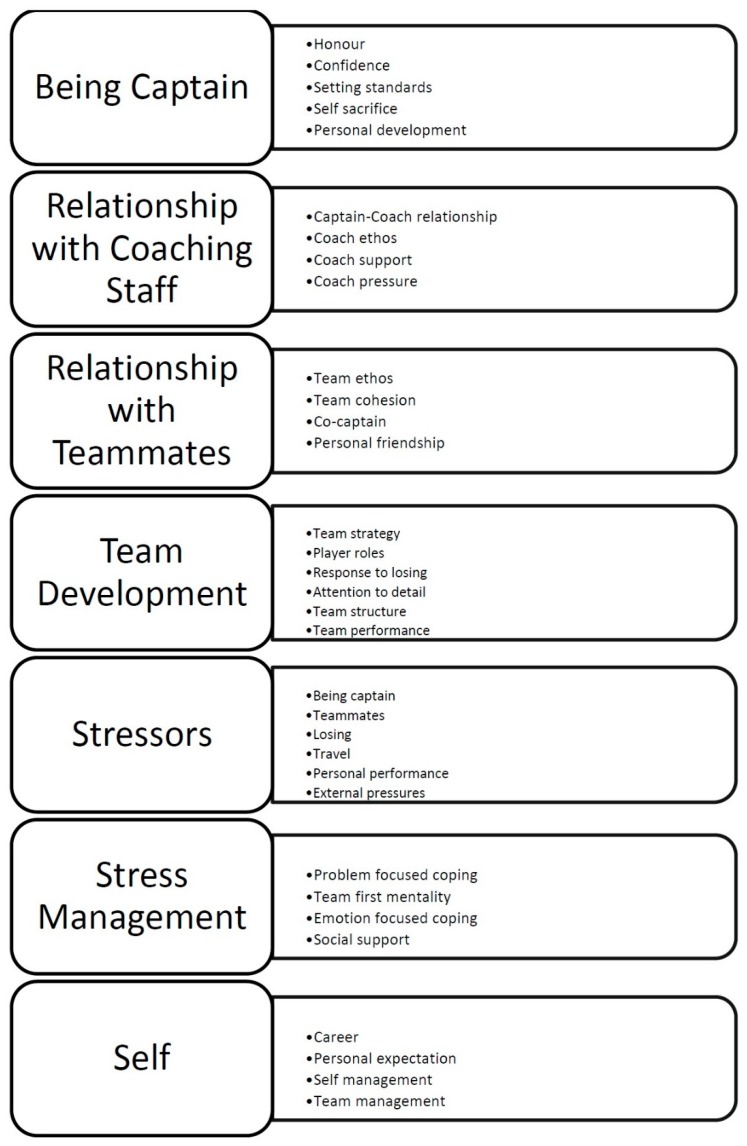
Higher-order and lower-order themes of captain’s reflection.

The data highlighted seven higher-order themes, which are used to form the basis of this discussion. These seven higher-order themes (Being captain; Relationship with coaching staff; Relationship with teammates; Team development; Stressors; Stress management; Self) have been identified from 33 lower-order themes (see Figure 1).

### 3.1. Being Captain

There is a distinct honour in being appointed captain of any team. It is also a position that provides a huge learning opportunity for any player. While previous experience may be of assistance, each new appointment is unique.

*“I always wanted to be captain. I’ve always wanted to be captain of this club. I felt I had already prepared myself to be captain… After the first month, I’ve really transitioned into the next stage with it… I’ve learned a lot about myself. I’ve learned a lot about individuals and I’ve learned a lot about team environments and how I approach things.”*

There are also a number of responsibilities, both stated and implied, which impact the captain and their role within the team. A key responsibility is to constantly set the standards of behaviour for the team [2]. While winning, this is easier to achieve and encourage within the team, however, during a losing season this becomes more difficult but this is rarely discussed.

*“It’s not about you anymore. You need to put the team before yourself… It’s hard (dealing with the losing and individuality within the team), I’m not going to lie… As a captain I just don’t think that’s the right thing to do.”*

At a professional level, the captain has more responsibilities than in other sports teams [7].

As a captain there is also a great deal of self-sacrifice. The role requires that the captain always puts the team first. “It’s what you’ve got to do for the team. You try to do the right thing for the team… You have to put everything aside for the betterment of the team”. As a key face of a professional sport organisation, the captain will be required to have more media contact or attend appearances (both community and sponsor focused). Intense media scrutiny is known to be challenging within professional sport [3]. A normal individual within the team has more leniency here and this can be spread throughout all players, but with only one captain she is required to attend despite fatigue levels or personal situation.

### 3.2. Relationship with Coaching Staff

Developing a positive coach-athlete relationship is a major focus within most sports teams [14]. There was a long working relationship between the head coach and the captain, and they had spoken at length prior to the season regarding team culture and personal goals. “*I chatted a lot with (coach) in the first month and he gave me great feedback. I really had a great relationship and we were talking about a lot of things*”. However, as the season progressed and the number of losses increased, strains in the relationship occurred. The head coach also had a tendency to place an increased amount of pressure on the captain, using her as the funnel to the rest of the team. “*(Coach) was really stressed that week about losing and I think he put his negativity on us*”. As losing streaks continued, many within the organisation became insular and began searching for solutions, not always with the whole team focus in mind. There was a perceived lack of accountability, and the captain acknowledged this increased pressure on the relationship between her and the coaching team.

*“Maybe (coach) or (assistant) needed to be harder on people. When (accountability) doesn’t come from coaches then it’s just not going to be… It’s hard when you are harping on about it and you’re not getting help from higher up.”*

During these losing streaks the coaches tried to implement a number of tactical changes, the result of which seemed to confuse the team and put more strain on the relationship. “*[The players] didn’t really know what was going (with tactical changes) or why (coach) was doing what he was doing*”. Open and honest communication between coach and captain is important because these individuals are the biggest leaders in the team [7]. The captain in this situation could not understand the purpose of the tactical changes, yet her role was to assist with information clarification and reinforcement, and to support the coach’s message.

Positively, while the team struggled to find a result, the ethos of the team stayed optimistic. “*The coaching staff stayed really positive… they were also really good while dealing with personnel changes*”. While admitting the situation affected her, as captain she still wanted to help the team and the coach. “*When things weren’t going well, I just did whatever I had to do for him. I back every decision that (the coaches) made*”.

### 3.3. Relationship with Teammates

Of primary concern for the captain was developing a strong team ethos and belief in the team’s ability. This was particularly true during the pre-season.

*“I kept saying we are such a talented group, we are going to be so hard to beat. Lots of us that played (previous season) really wanted to make amends and prove to the league that we were better than we had shown.”*

There were challenges as the season progressed, with one player failing to adhere to the team culture. “It was a struggle to get (player) to do an appearance and I just think that is not good enough. But, no one ever came to training and didn’t want to work hard”. A change in playing personnel helped the captain to develop this team ethos. “(New recruit) has been such a great person. She works hard, so she really brought a good energy”. The captain was hindered in her ability to influence the team by the loss of her co-captain to injury prior to the season commencing. Shared leadership has been seen as a significant component for team success [15]. The selection of co-captains was planned to allow for the strengths of each player to work in harmony. By nature, this captain is a more direct enforcer personality, while her co-captain is more empathetic. Without having that support there regularly affected her natural approach. “I have so much time for (co-captain). She is a very calming person… We have different ways we work… (co-captain) being injured probably impacted me quite a bit”. Social network analysis with sports teams has demonstrated how different individuals influence others [4], but how the removal of one network affects the whole team needs further analysis.

Team cohesion plays an important part to team success and engagement [16]. How regular losses affect this has not been investigated, but the captain stated how the situation actually helped the team connection.

*“The girls, off the court, we started to really bond around that (losing streak). We tried to stick together… I think we really bonded and we really got around each other… (coach) was probably a little bit negative and we just all thought we just have to stick together… We just have to stay in this or else this is going to be a nightmare.”*

The team were rewarded for their ability to be cohesive and celebrated a win together. “That night we all went out together and everyone came and everyone made an effort”. In amongst this the captain noted that the situation made her form some strong personal friendships.

*“It probably took me a while to warm to each individual and get around to each individual, but, I think, slowly I did. Obviously there’s people that you get to know quicker than others… I really enjoyed the group of girls and off the court, I’ve made life-long friends.”*

This social cohesion is different for a captain as opposed to a regular team member [17]. As a captain there can be a need to keep some distance to team members to allow for unpopular, but effective, leadership decisions to be made.

### 3.4. Team Development

Despite a positive start to the season, on reflection, the captain noticed a number of instances that would become issues. While winning, most of these cracks can be papered over, however, as losing streaks continue they become bigger and have greater influence.

*“We were playing good basketball, but I think those little attention to detail things defensively that have hurt us the whole season… I think accountability is a big thing for me, but people weren’t made accountable.”*

Losing is difficult for everyone. The coaching team initially tried to keep the same game strategy and aimed to improve the execution, but as the losing streak continued, constant changes were implemented. The impact of this had negative consequences, with the whole team losing continuity. Trying to push performance standards and supporting both the coaches and teammates, while learning new strategy, was difficult for the captain. “*Things just changed too much. We had a different kind of offence every week, and as a player that’s really hard*”. The effect of this may have compromised her ability to perform the role of captain.

The response to losing was positive throughout the season, however, as it became clear the team would not make finals, confidence diminished and individualism became evident in the team. The structure of the league exasperates this, as players are not assured of future playing contracts and some need to use the final games of the season to put themselves in the shop window to gain future opportunities within the sport. For the captain there is potentially more to lose, with not just her basketball performance but also her leadership skills under scrutiny.

*“(Losing) definitely did knock some confidence. We just forgot how to win…We only had one big blowout. Most of the games were pretty competitive. I think we all tried to stay positive… I think people try to do their own thing at this time of year, especially when we’re not playing for finals. If we were playing for finals it’s more about the team, but it’s like, well, it’s not really about the team anymore so I’m going to do what I want to do… That’s definitely what I felt. (The team attitude was) I don’t really care. I’m just going to go and shoot the ball and it’s not going to make an impact on our win-loss record anymore; it doesn’t matter.”*

This poses an interesting question as to whose responsibility is it to hold the team together.

### 3.5. Stressors

A number of stressors are present for any captain of any team [18], but these appear to be amplified during a losing season. As the captain tends to be one of the key faces of the organisation, just being captain is difficult during the losing streaks.

*“I guess everything when you’re captain is looked at. Three people can have a bad training, but I can’t… it doesn’t matter what’s going on, you just have to put on a brave face and then walk out and deal with it.”*

This suggestion that there are higher expectations on the captain is of interest. It is supported by the ideology of the captain needing to lead by example [3].

The influence of losing regularly increases potential stressors for the captain, particularly away from the performance environment [18]. Continual travel across the country became a burden and caused more fatigue.

*“We were on the road a lot. I felt like we were on the road for the whole of November, and that was tough… Those teams had won games so they had momentum, where we didn’t… A lot of teams had a two week break (over Christmas and New Year), but we only had four days.”*

With this stress came decreases in performance, which caused a debilitating negative spiral in confidence and mood. “(Losing and travel) probably knocked my confidence, which is hard because you are trying to be positive for the team”.

The stress is further complicated by the structure of the league and a need for players to consider their future. There are limited financial incentives and players tend to find other leagues to play in after the WNBL finishes; with the hope of performing well enough to gain a contract for the following WNBL season.

*“I think there was probably a couple of weeks where I had no idea what I was doing and you’ve got people asking you (about the off-season and next season)… Because statistically it wasn’t the season that I planned on having and I didn’t have the impact I wanted to make… so that was probably the hard thing for me, because I didn’t really know what I was doing… then you get an offer and you agree to it and then you don’t get a contract for two weeks.”*

The ability to continually perform and lead by example during all this uncertainty is difficult. If a team is winning, the momentum and determination to achieve can minimise this stress. How this experience would differ in well-funded sports, or in teams where contracts extend beyond one season (which is atypical in this league), would be of interest to compare.

### 3.6. Stress Management

*“Every day is a new day and you just have to remember that every day is a new day in terms of playing, and sometimes you can get bogged down in not playing well, or not shooting well or that type of thing, but guess you’ve just got to remember that things change from day-to-day and nothing is set in stone.”*

Emotion-focused coping was central to the management of stress throughout the losing season, with benefits for regulating emotional arousal and distress [19]. The use of emotion-focused coping is recognised to have greatest benefit when situations are outside of an individual’s control [20]. Therefore, goal disengagement and reengagement helped the captain to refocus after the original goal (making finals) had become unobtainable.

Regular reference was made to the need to *“keep working hard”* and the use of problem-focused coping strategies to help manage stressful situations. During the long losing streaks, the captain’s focus was to improve her own performance as a way of leading by example and demonstrating the team-first mentality that had been discussed pre-season.

*“You can say you try not to focus on yourself, but at the end of the day I have to focus on what I’m doing, because that’s what is best for the team… I just kept trying to work hard. I know deep down that I was doing all I could to improve and get better… Some things don’t always go your way but it’s just a learning experience every time.”*

Again, there are clear connotations of leading by example.

Social support is important for the management of stress no matter what the circumstance [19]. The effect of losing meant the provision of support shifted through the season for the captain. Initially it was from the coaching team and focused on performance, but progressed to more active coping assisted by the team sport psychologist, and emotional control in conjunction with friends and family. The effectiveness of each individuals support is not measured, but the use of various support networks appears to assist with the management of stressful situations [21].

*“(Coach) probably called me every second day to chat about things, and that was hard—it wasn’t hard, but it was probably just a transition and dealing with it in a different way… As the season went on I spoke with (team psychologist)… I spent a lot of time with family, just to take my mind off it. Just realising there was more to life.”*

### 3.7. Self

There is a fine balancing act for a captain in professional sports organisations between personal and career development, and the requirements of the team. Pre-season these can easily be intertwined i.e., a good personal performance will benefit the whole team performance. However, during a losing season there are a number of conflicts that can occur. For a captain there is a strong possibility that poor performance by the team will reflect badly on them directly. There is a potential fear that they can be viewed as a less accomplished performer. With the need to think about future career opportunities, many players may become insular or selfish on the court. An effective captain cannot.

Self-management and personal expectation play an important role throughout the season [17]. When losing, it takes a strong mindset to continually stay positive throughout. For the captain, as a key leader of the team, it is not feasible to show any negativity as this can have a major impact on the team. “*I always made sure I came to practice with a really positive mindset… For me just a mindset of going out and just being the best player you can be every time*”. This recognition to continually stay positive is interesting, as there is limited research discussing strategies and approaches to assist sports teams when going through these tough times. There may be a need to look at organisational psychology to provide some guidance. After all, “*Basketball is my job*”.

## 4. Discussion

The purpose of this study was to provide insights into the experience of the captain of a professional women’s basketball team during a losing season (i.e., the team won only 25% of games during the season). To date there has been minimal research into the experiences of captaincy, particularly in women’s professional sport, and no previous study has investigated the challenges and pressures that are placed on a captain when team performance is below expectation. Therefore, the findings provide a unique reflection into this phenomenon. Of prime interest is the need for the captain to wear multiple hats. They are the conduit between the coach and the team. The coach can place greater pressure on the captain to drive performance standards, at the same time the captain must demonstrate positive leadership skills to the rest of the team and be an ever-present face of the organisation. The captain needs to support the tactical and team structure decisions made by the coach, even when questioning their own performance. This increases stress and pressure placed upon her or him, which is different to a regular team member. Similarly, the coach can begin to distance themself from the team and become insular, looking for answers to break losing streaks, but the captain cannot. Team members turn to the captain for practical and emotional support and guidance, especially if she or he has been criticised by the coach.

The captain is one of the key faces of the professional sports organisation. There is greater responsibility and requirement to be available to the media, attend corporate sponsor functions, and be involved in promotional activity for the club. During a losing season these pressures intensify, as all these groups are looking for the captain to provide answers or they provide unsolicited advice regarding what the team “should” be doing. Having positive social support networks available is important to manage personal performance and well-being during losing periods. Even though the WNBL is a professional sports league, resources are limited. In this case the captain had a sport psychologist available who she could turn to for independent support, but many of the teams do not have this luxury. Further research is needed into athlete leadership and the formal roles captains occupy. While research has highlighted the benefits of shared leadership within sports teams, there are still a number of unique pressures and duties required of the captain. Provision of specific education programs on captaincy, for both the captain and the coach, may improve the effectiveness of both roles. Social support networks are also important to help manage the stressors of being captain in order to provide both practical and emotional support to her or him.

## 5. Conclusions

The purpose of the current study was to explore the experiences of a captain during a losing season. The impact of regularly losing and the recognition of unobtainable goals has a negative impact, but as a captain it is not acceptable to allow this frustration to show. A role of the captain is to galvanise the team to encourage peak performance and put aside any personal concerns for the sake of the team. The captain is a key face of the whole organisation and they are expected to act in a certain manner. It is important for support to be provided for a captain to assist management of their emotions and to minimise any negativity for the rest of the team. Winning covers many negative components in sport, but this is not an option for all teams. The selection, education, and development of captains needs more research, especially with the captain deemed as an important leadership role.
